# Lower thyroid stimulating hormone concentrations linked to suicidal ideations among individuals with anxiety and mood disorders

**DOI:** 10.1192/j.eurpsy.2023.1263

**Published:** 2023-07-19

**Authors:** V. Liaugaudaitė, A. Podlipskytė, J. Burkauskas, N. Mickuvienė, V. Adomaitienė, E. Zauka, V. Steiblienė

**Affiliations:** 1 Laboratory of Behavioral Medicine, Neuroscience Institute, Lithuanian University of Health Sciences, Palanga; 2 Clinic of Psychiatry, Lithuanian University of Health Sciences, Kaunas, Lithuania

## Abstract

**Introduction:**

Suicidal behavior is quite common in individuals with anxiety and mood disorders (AMD). One of the coexistence factors in suicidal behavior is thyroid dysfunction, however the results are still controversial (Shen et al. J Affect Disord 2019;(1)180-185; Zhou et al., Transl Psychiatry. 2021;11(1):97). The role of thyroid function in suicidal ideation among individuals with comorbid AMD have not been thoroughly investigated.

**Objectives:**

The aim of this cross-sectional study was to identify potential associations between thyroid function and suicidal ideation in a sample of individuals with anxiety and mood disorders.

**Methods:**

This exploratory study comprised 77 consecutive individuals with AMD (age range 18-73 years, 76% were females) attending the Psychiatry Day care unit. All individuals have been evaluated for current psychiatric diagnoses, suicidal ideation using the Mini International Neuropsychiatric Interview [M.I.N.I. 7.0.2]) as well as for socio-demographic factors and for current psychotropic medication use. Severity of depression and anxiety symptoms have been evaluated using the Patient Health Questionnaire-9 (PHQ-9) and the General Anxiety Disorder-7 (GAD-7). The biochemical blood tests were performed for the concentrations of thyroxine (FT4), triiodothyronine (FT3) and thyroid stimulating hormone (TSH). The univariate and multivariable logistic regression analyses were used to assess the association between biochemical parameters and suicidal ideation.

**Results:**

Of all study individuals with AMD – 56% have been identified as having current SI. There were not significant differences according to age, gender, education, BMI, smoking, depression and anxiety symptoms and current psychotropics use between SI and non-SI individuals. Serum FT4, FT3 and TSH concentrations were within normal range. However individuals with SI had significantly lower TSH concentrations in comparison to the non-SI (1.54 (0.77) vs. 2.04 (1.22) IU/L, respectively; p = 0.049), without significantly differences in FT4 and FT3 concentrations. A multiple logistic regression, adjusting for sociodemographic factors and severity of mental symptoms revealed, that non-SI individuals with AMD were likely to have higher TSH levels than SI (odds ratio = 2.15 (95% CI 1.10–4.22; p = 0.027).

**Conclusions:**

Among individuals with AMD, lower levels of TSH concentrations have been associated with presence of suicidal ideation, independently of sociodemographic factors and severity of depression and anxiety.

**Disclosure of Interest:**

V. Liaugaudaitė Grant / Research support from: European Union (project No P-PD-22-150) under the agreement with the Research Council of Lithuania (LMTLT)., A. Podlipskytė: None Declared, J. Burkauskas Consultant of: Cronos, N. Mickuvienė: None Declared, V. Adomaitienė: None Declared, E. Zauka: None Declared, V. Steiblienė: None Declared

